# Reliance of Host Cholesterol Metabolic Pathways for the Life Cycle of Hepatitis C Virus

**DOI:** 10.1371/journal.ppat.0030108

**Published:** 2007-08-31

**Authors:** Jin Ye

**Affiliations:** University of British Columbia, Canada

## Abstract

Hepatitis C virus (HCV), a single-stranded positive-sense RNA virus of the Flaviviridae family, infects more than 170 million people worldwide and is the leading cause of liver failure in the United States. A unique feature of HCV is that the viral life cycle depends on cholesterol metabolism in host cells. This review summarizes the cholesterol metabolic pathways that are required for the replication, secretion, and entry of HCV. The potential application of drugs that alter host cholesterol metabolism in treating HCV infection is also discussed.

## Introduction

Hepatitis C virus (HCV) exacts a heavy toll on public health. Approximately 3% of the world population is infected persistently with HCV [1]. According to the Centers for Disease Control and Prevention, 4.1 million Americans (1.6% of the United States population) are estimated to be infected by HCV, 3.2 million of whom become chronically infected [2]. These individuals account for most of the cases of liver failure in the United States [3]. Owing to its genomic variation, HCV variants are divided into six genotypes (genotypes 1–6), which differ from each other by 31% to 33% at the nucleotide level [4]. Genotype 1 HCV is the most prevalent viral variant found in the United States. This genotype is also less sensitive to the current interferon-based therapies, with only about 50% of patients responding to such treatment [5]. Thus, new therapeutic strategies to combat HCV infection are needed.

HCV is a single-stranded positive-sense RNA virus of the Flaviviridae family [6]. The 9.6-kilobase HCV genome encodes a single polyprotein that is post-translationally processed into at least ten structural and nonstructural (NS) proteins [7]. The amino-terminal third of the polyprotein encodes the virion structural proteins: core, E1, and E2, which are followed by a small integral membrane protein p7 that functions as an ion channel [8]. The remainder of the genome encodes the nonstructural (NS) proteins NS2, NS3, NS4A, NS4B, NS5A, and NS5B. Except for NS2, all of the other NS proteins are required for efficient viral RNA replication. These NS proteins (NS3–NS5B) form a viral replication complex on intracellular membrane vesicles, leading to a structure termed the membranous web [9,10].

For reasons still unknown, most clinically isolated HCV is difficult to replicate in cultured cells [7]. Thus, cells harboring HCV subgenomic replicons are widely used to study HCV replication. These replicons consist of HCV RNA engineered to express a selectable marker gene, *neo,* in place of the structural protein-coding region. A heterologous viral internal ribosomal entry site is inserted after the neomycin resistance cassette to direct translation of viral NS proteins (NS3–NS5B). When human hepatoma Huh7 cells were transfected with HCV replicon RNA and selected with G418, a cell line was established in which HCV RNA was constantly replicated [11]. This system, although very effective in studying HCV replication, is not able to produce infectious HCV particles. Recently, a strain of genotype 2 HCV was shown to be capable of replicating in Huh7 cells and producing HCV particles that are infectious to cultured cells [12–14]. The HCV particles produced from cell culture (referred to as HCVcc) were able to establish long-term infections in chimpanzees and in mice containing human liver grafts [15]. Moreover, virus recovered from these animals was still infectious in cell culture [15]. For reasons still unknown, only this strain of HCV or its derivative can be cultured in Huh7 cells to generate infectious viral particles.

**Figure 1:**
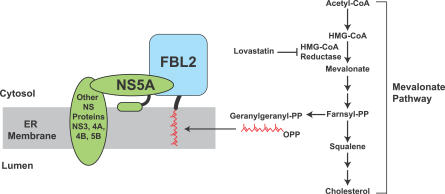
Requirement of the Mevalonate Pathway for HCV Replication Cells are capable of synthesizing cholesterol through the mevalonate pathway. This pathway also produces geranylgeranyl lipid, which is attached to the COOH-terminus of FBL2. Geranylgeranylated FBL2 binds to NS5A, an interaction required for HCV RNA replication. Lovastatin, an inhibitor of HMG CoA reductase, blocks the entire mevalonate pathway. As a result, cells are depleted of geranylgeranyl lipid and replication of HCV is inhibited. PP, pyrophosphate.

HCV infection is mainly restricted to hepatocytes [1], which play a vital role in mammalian cholesterol homeostasis. Like all mammalian cells, hepatocytes are able to acquire cholesterol through two pathways. Cholesterol can be synthesized from acetyl-CoA via the mevalonate pathway [16], which also generates several isoprenoids, including farnesyl and geranylgeranyl lipids that are covalently attached to the COOH-terminus of certain proteins [17] (Figure 1). Cells can also acquire low density lipoprotein (LDL)–associated cholesterol in serum through LDL receptor–mediated endocytosis [18]. The LDL receptor binds to LDL particles, which are internalized via the clathrin-dependent pathway of receptor-mediated endocytosis. Following endocytosis, the lipoprotein is degraded in lysosomes and cholesterol in LDL is released into the cells [18].

In mice, hepatocytes can also acquire a substantial amount of cholesterol through scavenger receptor class B type I (SR-BI)–mediated uptake of cholesterol from high density lipoprotein (HDL) particles [19]. In humans, hepatic cholesterol contributed by HDL is not as significant. This is because humans, but not mice, express cholesteryl ester transfer protein, which transfers cholesterol from HDL to LDL or its precursor, very low density lipoprotein (VLDL) [20]. As a result, the amount of HDL-associated cholesterol is much lower in humans compared to mice, in which HDL particles transport most of the plasma cholesterol.

**Figure 2:**
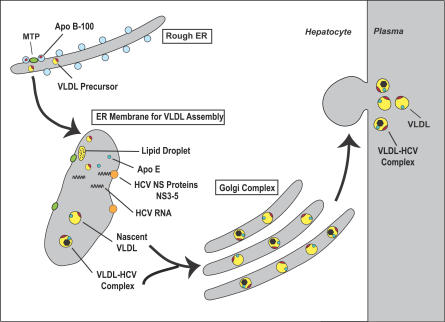
HCV Replicates on ER Membranes Involved in the Assembly of VLDL and Is Secreted Together with VLDL The assembly of VLDL begins with the synthesis of apoB in the rough ER, resulting in the formation of a VLDL precursor that contains only a small amount of lipid. In the lumen of the ER, this precursor is fused with lipid droplets (enriched in triglyceride and cholesterol) to generate nascent VLDL. This reaction is mediated by MTP. The nascent VLDL particles, which contain both apoB and apoE, are secreted into plasma through exocytosis. The ER membranes involved in the assembly of VLDL are also enriched in HCV NS proteins and RNA. Replication of HCV on these membranes might allow the virus to attach to or become incorporated into VLDL so that HCV is secreted together with VLDL.

Hepatocytes play a crucial role in regulating mammalian cholesterol metabolism by exporting cholesterol together with triglycerides through secretion of VLDL (Figure 2). The assembly of VLDL begins with synthesis of full length apolipoprotein B (apoB), a 540-kDa protein that confers structural integrity to VLDL [21]. Nascent apoB is then fused with lipid droplets that are rich in triglyceride and cholesteryl esters in the lumen of the endoplasmic reticulum (ER). While most cells contain cytosolic lipid droplets, hepatocytes, as well as other cells producing lipoprotein particles that contain apoB, have lipid droplets in the lumen of the ER [22]. Fusion between lipid droplets and apoB requires the activity of microsomal triglyceride transfer protein (MTP) [23]. Without MTP-mediated lipid transfer, the secretion of apoB is blocked and the protein is degraded in cells [24]. The importance of MTP in VLDL assembly is demonstrated by the observations that genetic defects in MTP severely reduce VLDL secretion [25,26]. The resulting VLDL particles are composed of a hydrophobic core of triglycerides and cholesteryl esters surrounded by a surface coat containing phospholipids, free cholesterol, and two predominant lipoproteins, apoB and apolipoprotein E (apoE). VLDL is secreted out of cells through exocytosis [27].

Nascent VLDL particles released into plasma are not ligands for the LDL receptor [28]. These lipoprotein particles are substrates for lipoprotein lipase, which hydrolyzes the triglycerides in the core of the lipoprotein particles [29]. A large proportion (∼70%) of the resulting particles, called intermediate density lipoprotein (IDL) particles, is efficiently removed from plasma by the LDL receptor on hepatocytes [18]. The remaining IDL particles in the circulation are converted to LDL particles by a reaction catalyzed by hepatic lipase, which further reduces the amount of triglycerides in the lipoprotein particles [30]. Once formed, LDL is taken up by the LDL receptor on hepatic as well as nonhepatic cells [18].

## Requirement of the Mevalonate Pathway in HCV RNA Replication

The first clue that the mevalonate pathway is involved in HCV RNA replication was the finding that treatment of cultured cells with lovastatin, a cholesterol-lowering drug that inhibits 3-hydroxy-3-methylglutaryl coenzyme A (HMG CoA) reductase, the rate-limiting enzyme in the entire mevalonate pathway [16], inhibited HCV RNA replication [31]. Similar results were subsequently obtained by others [32–34].

The product of the mevalonate pathway required for HCV RNA replication turns out to be a geranylgeranyl lipid, as shown by experiments in which the inhibition of HCV RNA replication by lovastatin was overcome by the addition of geranylgeraniol, but not farnesol or cholesterol [31,32]. Geranylgeranyl serves as a lipid substrate for protein geranylgeranylation, a post-translational modification that covalently attaches geranylgeranyl to various cellular proteins to facilitate their membrane association [17]. Thus, it appears that one or more geranylgeranylated proteins are required for HCV RNA replication. This notion is further supported by the observation that HCV replication could be blocked by an inhibitor of geranylgeranyl transferase I [31], an enzyme that transfers geranylgeranyl groups to cellular proteins [17].

Inasmuch as HCV does not encode a viral protein that contains the signature COOH-terminal C*AA*X motif (i.e., cysteine-X-X-leucine or isoleucine, in which X is an aliphatic amino acid) that specifies geranylgeranylation, it was hypothesized that a geranylgeranylated host protein may be required for HCV RNA replication. In this regard, a 50-kDa geranylgeranylated host protein was found to co-immunoprecipitate with the viral protein NS5A [35]. With the knowledge of the molecular weight of the protein and the sequence motif that specifies geranylgeranylation, this protein was identified as FBL2 by a bioinformatics approach [35]. Geranylgeranylation of FBL2 appears to be critical for HCV replication because the association between FBL2 and NS5A depends on geranylgeranylation of FBL2, and this interaction is required for HCV RNA replication [35] (Figure 1). The exact role played by FBL2 in HCV RNA replication remains unclear because the physiological function of FBL2 is not known. FBL2 contains an F-box, a domain known to be involved in protein ubiquitination [36]. Thus, it is possible that protein ubiquitination may play a role in HCV RNA replication. It should be noted that all of the aforementioned studies used genotype 1 HCV replicons. It is not known whether geranylgeranylation of host proteins is required for replication of other genotypes of HCV.

Unlike geranylgeranyl, the requirement of cholesterol for HCV RNA replication is still under debate. The observation that addition of cholesterol failed to rescue HCV RNA replication in the presence of lovastatin suggests that cholesterol is not directly involved in HCV RNA replication [31]. However, differing results were obtained when cells were depleted of cholesterol by treatment with methyl-β-cyclodextrin, which directly extracts cholesterol from cell membranes [37]. Under these conditions, HCV RNA replication is either not affected [38] or reduced by 50% [39]. The inconsistency in these results could arise from a secondary effect of general cellular toxicity that occurs when cells are depleted of cholesterol, or from a difference in the HCV genotype used in these studies.

## Requirement for VLDL Assembly in HCV Secretion

Like all positive-strand RNA viruses, HCV RNA replication occurs in association with cytoplasmic membrane vesicles. In the case of HCV, these structures (membranous webs) have been visualized in cultured human hepatoma Huh7 cells that harbor a subgenomic replicon of HCV [9,40]. Recently, membrane vesicles containing the HCV replication complex from Huh7 cells that harbor an HCV replicon were isolated [41]. Proteomic analysis revealed that these vesicles are enriched in apoB, apoE, and MTP, proteins known to be required for the assembly of VLDL [41]. Interestingly, VLDL synthesis is not required for HCV RNA replication [41]. This result is consistent with the previous findings that HCV RNA can replicate in HeLa and HEK-293 cells [42–44], which do not produce VLDL. The reason for co-localization of the HCV replication and VLDL assembly appears to lie in a requirement for co-assembly or secretion of VLDL and HCV particles (Figure 2). Thus, When Huh7 cells that constitutively produce infectious HCV [45] were treated with an MTP inhibitor or siRNA targeting apoB, the secretion of VLDL and HCV were both inhibited [41].

Although VLDL secretion is required for HCV production in cultured cells, this requirement has not been demonstrated in HCV-infected patients. However, it was shown that HCV particles circulating in serum are in complex with VLDL [46–48]. Thus, it appears that HCV particles are attached to or incorporated into VLDL during the assembly of the lipoprotein particles and secreted together with VLDL. Although the nature of the association between HCV and VLDL remains unclear, studies with HCV isolated from infected patients suggests that HCV may reside in the lipid-rich core of VLDL, since delipidation of lipoprotein particles that contain apoB is required to observe the capsid-like structures of HCV [46,47]. If HCV indeed hides in the core of VLDL as suggested [49], it makes the virus unique in that the entire virion is not exposed to serum during circulation. If this model is correct, it will also predict that HCV has to escape from VLDL-derived lipoprotein particles during viral entry so that the structural protein E2 can interact with its cellular receptors, a step required for HCV RNA to enter cytosol (see below). Exactly what triggers the release of HCV from the lipoprotein particles is not known. If LDL receptor–mediated endocytosis is required for HCV entry (see below), the virus is likely to escape VLDL-derived particles in endocytic vesicles after the lipoprotein particles are disassembled during endocytosis. This scenario does not necessarily contradict the observation that entry of HCVcc was inhibited by antibodies targeting viral structural protein E2 [13], since these antibodies might also be included in endocytic vesicles that contain HCV. It was reported previously that immunoglobin G is able to enter clathrin-coated pits nonspecifically through fluid-phase endocytosis [50,51]. Thus, these antibodies may block HCV entry by binding to the viral structural protein after the virus is released from the lipoprotein particles in endocytic vesicles.

The observation that HCV replicates on membrane vesicles involved in the assembly of VLDL might also help to explain the effect of HCV infection on hepatic release of VLDL. Infection by HCV, particularly the genotype 3 variant, leads to a reduced rate of VLDL secretion [52,53]. The reduction in VLDL secretion could arise from inhibition of MTP by viral proteins. It has been reported that mRNA levels of MTP are reduced in Huh7 cells harboring HCV replicons [54], and that transgenic mice expressing HCV core protein in livers have reduced MTP activity [55]. However, the reduction in MTP activity has not been demonstrated in cells or patients that are infected by HCV. The role of reduced VLDL secretion in HCV infection is currently unknown. It is possible that by delaying the secretion of VLDL, HCV might have enough time to replicate and assemble into VLDL.

Besides liver, intestine is the only other tissue that produces apoB-containing lipoprotein particles. These particles, called chylomicrons, contain a shorter form of apoB [56]. Although HCV infection in intestinal cells was reported in a fraction of HCV-infected patients [57,58], the amount of HCV detected in intestine was much lower than that in liver [58]. However, in patients that had HCV infection in intestine, HCV particles circulating in serum were in complex with chylomicrons [59]. Thus, it appears that intestinal cells are able to export HCV, but they might not contain cellular factors that support efficient entry and/or replication of HCV.

## Requirement of Lipoprotein Receptors for HCV Entry

HCV enters cells through multiple cellular receptors. CD81, which directly binds to HCV structural protein E2 [60,61], is required for HCV to enter host cells [13,62]. Interestingly, the amount of CD81 expressed on the cell surface is affected by cellular cholesterol content. When cells were depleted of cholesterol by treatment with methyl-β-cyclodextrin, the amount of CD81 located on plasma membrane was reduced, resulting in a reduction in HCV entry [38]. Recently, claudin-1 was identified as a receptor functioning at a step later than CD81 [63]. Both CD81 and claudin-1 have been demonstrated to be required for HCV entry by experiments showing that viral entry was blocked in cells that do not express either one of these proteins, and that reintroduction of the protein into the cells specifically restores the viral entry [13,63]. These receptors appear to act at later steps of HCV entry, because CD81 is not required for HCV to bind to the cell surface [64] and is not known to initiate the clathrin-mediated endocytosis that is required for entry of HCV [65]. Considering the association between HCV and VLDL-derived lipoprotein particles, lipoprotein receptors might function at earlier steps in HCV entry.

The LDL receptor, which plays a predominant role in acquiring VLDL-derived lipoprotein particles through clathrin-mediated endocytosis in hepatic cells [66,67], has been reported to be involved in HCV entry. These studies demonstrated that cellular binding or uptake of HCV particles isolated from infected patients correlated with LDL receptor activity on the cell surface [47,68–70]. In contrast to the results with genuine HCV isolated from serum of HCV-infected patients, HCV pseudo particles (HCVpp), which were assembled by displaying HCV structural proteins E1and E2 onto retroviral core particles, did not require the LDL receptor for their entry [71]. These results do not argue against an LDL receptor-mediated uptake of HCV since HCVpp were produced in HEK-293 cells that do not produce VLDL [71]. These results also suggest that virus-associated lipoproteins are required for LDL receptor–mediated binding of HCV.

Nevertheless, the role of the LDL receptor in HCV entry is still uncertain, because cellular binding or uptake of HCV in all of the aforementioned studies did not result in viral infection. This outcome could be explained by the difficulty of most clinically isolated HCV to replicate in cultured cells [7]. However, the possibility that LDL receptor–mediated viral entry does not lead to viral infection cannot be ruled out. More studies using HCVcc that effectively infect cultured cells are needed to demonstrate the requirement of the LDL receptor in HCV entry.

Mutations disrupting the function of the LDL receptor produce autosomal dominant familial hypercholesterolemia, which affects 0.2% of the world population [72]. Affected individuals have elevated plasma levels of LDL cholesterol, which causes premature coronary atherosclerosis [72]. Mutations in LDL receptors might protect affected individuals from HCV infection, if the LDL receptor is indeed involved in HCV entry. An analysis comparing the frequency of HCV infection in people expressing normal LDL receptor versus those affected by familial hypercholesterolemia will be needed to address the question.

Another lipoprotein receptor that is implicated in HCV entry is SR-BI, which binds to HDL [73] and oxidized LDL [74,75]. The involvement of SR-BI in HCV entry is indicated by the observations that entry of HCVcc to cells was blocked by antibodies directed against SR-BI [38,76] and inhibited by excessive amounts of oxidized LDL [77]. However, it is not known whether HCV entry is blocked in cells that do not express SR-BI. Unlike with the LDL receptor, the interaction between HCV and SR-BI may not depend on lipoproteins associated with the virus because the HCV structural protein E2 directly binds to SR-BI [78]. This notion is further supported by observations that entry of HCVpp, which does not associate with VLDL, required SR-BI [79–82].

## Potential Application of Drugs That Target Cholesterol Metabolic Pathways in Treating HCV Infection

A major obstacle in combating HCV infection is that the fidelity of the viral replication machinery is notoriously low, thus enabling the virus to quickly develop mutations that resist compounds targeting viral enzymes [83]. Therefore, drugs targeting the host proteins required for HCV infection may be more effective in combating the viral infection. A unique aspect of HCV that has not been observed in other viruses is that the entire viral life cycle is associated with cholesterol metabolism in host cells. Thus, drugs that target cholesterol metabolism might be useful in treating HCV infection.

Treatment of cells with statins, the widely used cholesterol-lowering drugs, has been reported to inhibit HCV RNA replication [31–34] by depletion of geranylgeranyl lipids [31,32]. However, the doses of statins currently used to treat hypercholesterolemia by inhibiting cholesterol synthesis, thereby stimulating expression of the LDL receptor in the liver, are not high enough to inhibit the synthesis of geranylgeranyl lipid. Applying statins to treat HCV will require much higher doses and would likely cause toxicity in the liver and other organs.

Another class of drugs designed for treating hypercholesterolemia blocks the assembly and secretion of VLDL. These drugs may also be effective in treating HCV infection because they inhibit production of HCV particles from infected cells [41]. In this regard, antisense RNA drugs targeting apoB [84] and several MTP inhibitors [85,86] have already been tested in clinical trails because of their ability to block VLDL secretion, thereby lowering the plasma levels of VLDL triglycerides and LDL cholesterol. Long-term treatment with MTP inhibitors led to the toxic accumulation of fat in livers [85,86], thus hampering the approval of these drugs for the treatment hypercholesterolemia on a long-term basis. However, short-term treatment (up to several weeks) reduced the plasma level of VLDL with only minor adverse effects, which disappeared after drug removal [85]. It will be interesting to examine whether short-term treatment with MTP inhibitors is beneficial in treating HCV infection.

## Supporting Information

### Accession Numbers

The National Center for Biotechnology Information (http://www.ncbi.nlm.nih.gov/) accession numbers for human apoB, apoE, CD81, claudin-1, FBL2, LDL receptor, MTP, and SR-BI are NP_000375, NP_000032, NP_004347, NP_066924, NP_036289, NP_000518, NP_000244, and NP_005496, respectively. 

## Abbreviations

apo: apolipoprotein
ER: endoplasmic reticulum
HCV: hepatitis C virus
HDL: high density lipoprotein
HMG CoA: 3-hydroxy-3-methylglutaryl coenzyme A
IDL: intermediate density lipoprotein
LDL: low density lipoprotein
MTP: microsomal triglyceride transfer protein
NS: nonstructural
SR-BI: scavenger receptor class B type I
VLDL: very low density lipoprotein
